# Recent Advances in the Catalytic Conversion of Methane to Methanol: From the Challenges of Traditional Catalysts to the Use of Nanomaterials and Metal-Organic Frameworks

**DOI:** 10.3390/nano13202754

**Published:** 2023-10-13

**Authors:** Seyed Alireza Vali, Ahmad Abo Markeb, Javier Moral-Vico, Xavier Font, Antoni Sánchez

**Affiliations:** Composting Research Group (GICOM), Department of Chemical, Biological, and Environmental Engineering, Universitat Autònoma de Barcelona, Bellaterra, 08193 Barcelona, Spain

**Keywords:** methane oxidation, catalysis, global warming, nanoparticles, metal-organic frameworks, renewable energy, zeolite, methanol synthesis

## Abstract

Methane and carbon dioxide are the main contributors to global warming, with the methane effect being 25 times more powerful than carbon dioxide. Although the sources of methane are diverse, it is a very volatile and explosive gas. One way to store the energy content of methane is through its conversion to methanol. Methanol is a liquid under ambient conditions, easy to transport, and, apart from its use as an energy source, it is a chemical platform that can serve as a starting material for the production of various higher-value products. Accordingly, the transformation of methane to methanol has been extensively studied in the literature, using traditional catalysts as different types of zeolites. However, in the last few years, a new generation of catalysts has emerged to carry out this transformation with higher conversion and selectivity, and more importantly, under mild temperature and pressure conditions. These new catalysts typically involve the use of a highly porous supporting material such as zeolite, or more recently, metal-organic frameworks (MOFs) and graphene, and metallic nanoparticles or a combination of different types of nanoparticles that are the core of the catalytic process. In this review, recent advances in the porous supports for nanoparticles used for methane oxidation to methanol under mild conditions are discussed.

## 1. Introduction

Global warming has raised many concerns during the last few decades. Undoubtedly, the major cause is the release of greenhouse gases into the air [1,2]. Among such gases, methane and carbon dioxide make the biggest contribution to the global problem. Furthermore, when compared by mass, methane has around 25 times more effect on global warming than carbon dioxide. Hence, scientists have given a sharper focus on the conversion of methane to more beneficial chemicals, that is, higher hydrocarbons or liquid fuels [3].

The production of methanol, formaldehyde, propanol, and other compounds through various methods has been gaining more interest to unlink its production from non-renewable sources. So far, diverse studies have been carried out for the catalytic conversion of methane to syngas and methanol on different transition metals, including Ir, Pt, Rh, and Ru [4,5,6], perovskites [7,8] and single metal atoms incorporated in supports such as graphene [8], metal-organic frameworks [9,10] and metal oxides [11,12]. The conversion of methane into methanol is normally carried out through direct and indirect pathways. While through an indirect route, via a two-step procedure, methanol is formed by a catalytic reaction from syngas (CO + H_2_), which is produced via oxidation or steam reforming of methane, methane can also be directly converted to methanol through a direct route. Since steam reforming is a thermodynamically unfavorable reaction due to its intrinsic endothermic nature and therefore is immensely energy intensive, the indirect route may not be the best option, especially when it comes to industrial applications.

Thus, direct conversion of methane under mild conditions has recently become the main objective of researchers’ studies. Common direct pathways so far have been partial oxidation of methane (POM) to methanol and acetic acid, conversion of methane to olefins and aromatics through a non-oxidative route (NOCM), and oxidative coupling of methane (OCM). Whereas through OCM and NOCM routes other products rather than methanol are generated, the path that leads to a high methanol yield is stated to be partial oxidation of methane (POM), which is a thermodynamically favorable process since the change in Gibbs free energy for such reactions is negative using oxygen as the oxidant [13,14,15,16].

In general, there are several challenges to the direct conversion of methane to methanol. One is the strong C-H bond in methane, which requires severe conditions such as high temperatures to be cleaved. Due to high costs, this issue questions the industrial applicability of the process. Moreover, it causes the overoxidation of the produced methanol to produce more thermodynamically favorable products such as carbon monoxide and dioxide. The reason for this phenomenon is that the dissociation energy of the C-H bond in methanol is lower than that of methane. In other words, as the temperature increases, methanol is more susceptible to oxidation than methane. Consequently, the selectivity for the formation of methanol will decrease due to the generation of other products. In this respect, a catalyst that may activate the C-H bond of methane and simultaneously impede methanol oxidation would be of significant value [17,18]. In fact, methane monooxygenase enzymes existing in aerobic methanotrophic bacteria are naturally capable of converting methane to methanol under ambient conditions thanks to their intrinsic catalytic system [19]. Hence, emulating such a natural catalytic system for the conversion of methane to methanol has attracted researchers’ interest.

For this purpose, scientists have tried to take advantage of zeolite-based catalysts, metal-organic frameworks (MOFs), and graphene, which inherently have a large number of active sites as well as being perfect hosts for the incorporation of active sites, specifically those existing in nano-catalysts, resembling those found in the monooxygenase enzymes [20,21,22]. Such materials have gained interest for the catalytic conversion of methane to methanol in the last few years. One of the underlying reasons for the incorporation of nano-catalysts into porous media is to overcome a significant obstacle regarding these nano-catalysts high surface energy, which causes their aggregation and instability during the catalytic reaction and consequently their poor catalytic performance at short-medium times.

In this review, a quick revision of the significant factors in the catalytic conversion of methane to methanol (activation of the C-H bond in methane and its connection to methanol selectivity, reaction conditions such as temperature, pressure, and residence time) is presented. Afterward, a deep review of the recent studies that have taken advantage of three emerging supports—graphene; zeolite; and specifically MOFs—is developed; especially when they are doped with the proper nanomaterials. These emerging materials have been demonstrated to be the most competent candidates due to their properties, and they will be extensively presented and compared in terms of methanol yield and selectivity, as well as the conditions of these emerging catalytic systems such as temperature, pressure, and reaction time. Finally, a brief review of catalytic reactors is presented.

## 2. Conversion of Methane to Methanol Routes

### 2.1. Direct and Indirect Routes

**Figure 1:**
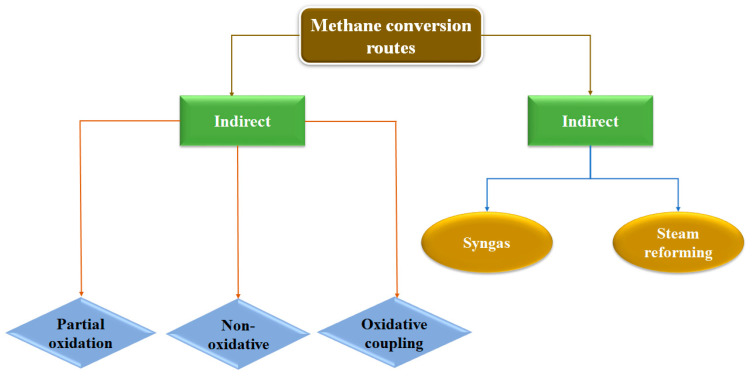
Routes for methane conversion.

As previously commented, the conversion of methane into value-added chemicals such as methanol, olefins, aromatics, and oxygenated compounds can be achieved through two different routes, as summarized in Figure 1. On one hand, the indirect route for methane to methanol conversion is a two-step process: (1) Partial oxidation or steam reforming of methane to syngas (CO + H_2_), and (2) catalytic conversion of syngas to methanol. It is known that the steam reforming step is an endothermic reaction (∆H^0^_298K_ = +206.2 kJ mol^−1^) with an operating temperature between 800 and 1000 °C. Therefore, the process is extremely energy-demanding. Hence, scientists have attempted to circumvent the intermediate syngas production step and directly convert methane at low temperatures. Partial oxidation of methane (POM) to methanol and acetic acid, conversion of methane to olefins and aromatics through a non-oxidative route (NOCM), and oxidative coupling of methane (OCM) are among the well-known direct routes for methane conversion reactions.

Partial oxidation of methane is an interesting energy-saving process that converts methane to profitable oxygenates such as methanol, formic acid, formaldehyde, and methanol precursors. This route, using oxygen as an oxidant, is thermodynamically more convenient to carry out (Equation (1)). NO and H_2_O_2_ can also be exploited as oxidants in POM [13,14,15,16]. However, thermal catalytic conversion of methane to methanol encounters major challenges, such as the activation of C-H in methane, which occurs at extremely high temperatures.

2CH_4_ + O_2_ → 2CH_3_OH ΔG^0^ _298K_ = −223 kJ mol^−1^ (1)

In addition to this, different works have been published regarding the use of zeolite-based catalysts that can contribute to the formation of methanol and acetic acid at low temperatures by activating methane and oxygen [23], although the reaction needs to be carried out at low methane conversion to preserve the target products from overoxidation.

Moreover, other studies have been aimed at addressing the challenges of the partial oxidation of methane to methanol. Some examples to overcome this phenomenon use different approaches such as the activation of methane in a liquid phase using H_2_O_2_ as an oxidant for the conversion of methane to methanol over copper-promoted Fe-ZSM-5 [24], a stepwise process for the conversion of CH_4_ over Cu-containing zeolite using H_2_O as oxidant [25], a new modified Au-Pd/zeolite catalyst for enhanced methanol productivity by in-situ generated hydrogen peroxide at low temperature (70 °C) [26], a hybrid system combining metal oxide (MO_x_)-coated glass beads as an alternative to thermal catalysis for the production of liquid oxygenates at atmospheric pressure and room temperature [27], a selective formation of methanol as unique oxygenate in a CO-assisted direct catalytic reaction over Cu-CHA zeolite catalyst [28], and the use of water for the mild oxidation of methane to methanol with high methanol selectivity over a gold single atom on phosphorous nanosheets under light irradiation [29].

On the other hand, NOCM (non-oxidative coupling of methane) is a promising route for the direct transformation of methane to hydrogen and ethane, despite the thermodynamically unfavorable nature of the reaction (Equation (2)):

2CH_4_ → C_2_H_6_ + H_2_ ΔG^0^ _298K_ = 68.6 kJ mol^−1^ (2)

As mentioned, OCM (oxidative coupling of methane) is another direct route for methane conversion. During this route through Equations (3) and (4), the methane is primarily converted to C_2_H_4_ and C_2_H_6_ in the presence of an oxidant (Equation (3)):

4CH_4_ + O_2_→ 2C_2_H_6_ + 2H_2_O DG^0^ _298K_ = −320.8 kJ mol^−1^ (3)

2C_2_H_6_ + O_2_→ 2C_2_H_4_ + 2H_2_O DG^0^ _298K_ = −254.9 kJ mol^−1^ (4)

As observed, the change in Gibbs free energy is negative, and this route is thermodynamically favorable. Regarding OCM and NOCM, many studies have been presented in the literature [30]. Hence, this review will be focused on the partial oxidation of Methane to methanol and will not further discuss the other routes.

### 2.2. Challenging Parameters in Methane to Methanol Catalysis

**Figure 2:**
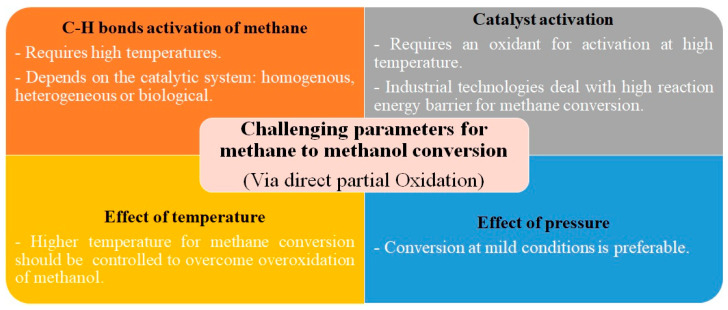
Challenges facing the methane activation and its conversion to methanol.

As previously commented, many catalysts have been developed and used for the direct partial oxidation of methane to methanol. However, there are several challenges regarding this catalytic process, such as the activation of the C-H bonds of methane, the need for catalyst activation, and the conditions of temperature and pressure necessary for acceptable methanol productivity and selectivity. In other words, developing a selective and efficient catalyst encounters a major challenge in the simultaneous control of the kinetics of methane transport, activation, hydroxylation, and the desorption and removal of methanol. All these issues will be discussed in this section. Such challenges are summarized in Figure 2 and will be discussed in this section.

#### 2.2.1. Activation of C-H Bonds and Its Connection to Selectivity

The activation of C-H bonds in methane requires high temperatures in traditional catalytic systems. However, under these conditions, the produced methanol can be overoxidized to produce thermodynamically more favorable products. In addition, the polar structure of methanol compared to the non-polar methane molecule contributes to the easier oxidation of methanol than methane since methanol molecules are more readily absorbed on the surface of the catalysts and activated for oxidation. Therefore, an ideal catalyst would be one that can facilitate methane activation and, at the same time, hamper methanol oxidation [17,18]. In this regard, a large number of strategies have been proposed in biological, homogenous, and heterogeneous catalytic systems. In nature, methane monooxygenase enzymes are present in aerobic methanotrophic bacteria that directly convert methane to methanol under ambient conditions due to their ability to control the transport of oxygen, methane, and protons to the active centers. Hydrophobic cavities linked together in the methane monooxygenase open the access gate to the oxygen and methane into the active center via the hydrophobic passage. Then, the activation of the oxygen in the metal center of the monooxygenase proteins leads to the formation of an oxidative intermediate that is able to perform the cleavage of the strong C-H bonds of methane [19]. When the enzymes rearrange their conformation, cavities dissociate from each other, resulting in the blockage of the hydrophobic passage and consequently restricting back diffusion and overoxidation of methanol while simultaneously opening separated hydrophilic pores for methanol to be removed. This biological system leads to an exceptionally high selectivity for methanol and can be an example of the control of mass transfer to and from the active sites. Therefore, it can be concluded that the presence of a hydrophobic cavity in the proximity of catalytic sites might lead to a higher affinity for methane than methanol [31]. However, such interesting ideas cannot be simply translated into simple homogenous catalysts [32]. In a homogenous catalytic system, the approach adopted is to functionalize methane in the form of a methyl ester that is more stable in this reaction environment. Afterward, this methyl ester is easily hydrolyzed for the recovery of methanol [33]. Regarding heterogeneous catalysis, the published studies have been focused on the investigation of materials that have a reactivity and a morphology resembling those found in methane monooxygenases. The exploitation of zeolite-based catalysts and the incorporation of different types of MOFs and graphene supports are among these attempts, and they will be discussed later.

#### 2.2.2. Activation of Catalyst

One of the principal challenges in methane conversion to methanol is that the reaction has a stoichiometry of 1:1 [34]. This has originated the so-called “stepped conversion” process. In this procedure, the catalysts are first activated with an oxidant at a high temperature and then exposed to methane to form methanol at a lower temperature. Finally, methanol is extracted using steam flow. In this way, methanol selectivity is higher since the catalyst is exposed to the oxidant and methane separately. However, there are inevitably considerable obstacles, such as the fact that industrial technologies need a high reaction energy barrier for methane conversion; therefore, there are energy-intensive processes when it comes to practical and industrial terms [35].

#### 2.2.3. Temperature and Pressure

Temperature and pressure are crucial parameters for methane oxidation to methanol in terms of the activation of catalysts and the cleavage of the methane C-H bond. In addition to the cost of having high temperatures, the issue of overoxidation of methanol at high temperatures is also noteworthy. A solution to achieve methanol formation at mild temperatures is the use of photocatalysts. As mentioned, the dissociation of the first C-H bond in methane is the rate-limiting step for methane activation. Under pure thermal conditions, extremely high temperatures will be required for the cleavage of the C-H bond [36]. Photocatalysis can be a potential solution to such a barrier. In photocatalysis, through the excitation of photons with high energies, active intermediates can be easily produced. Such active intermediates are capable of triggering the cleavage of the C-H bond at mild temperatures. This way, the sintering and agglomeration of active sites that usually happen due to the harsh reaction temperatures would be alleviated as well. Hence, by selecting the suitable photocatalyst, the high-temperature conversion of methane to methanol via the partial oxidation route would be feasible at low temperatures using solar energy. Photocatalytic partial oxidation of methane to methanol or formic acid can be carried out over oxide photocatalysts such as MoO_3_, VO_X_/SiO_2_ [14]. The active O species photogenerated on the surface of photocatalysts play a significant role in methane activation via cleaving the hydrogen from methane. Corma et al. [37] demonstrated that photoirradiation caused the dissociation of surface O-H bonds on the silica-zeolite. This resulted in the formation of Siloxyl radicals, which were capable of generating methyl radicals from methane. As far as the photocatalysis of methane is concerned, the oxidant agent is of importance. For instance, Anpo et al. [13] revealed that using nitric oxide (NO) instead of molecular oxygen as the oxidant and upon UV light irradiation over vanadium oxide immobolized on MCM-41 at ambient temperature, methanol can be formed with much higher selectivity, whereas overoxidation of methanol occurs in the presence of oxygen as the oxidant. Xie et al. [16] observed that when an appropriate amount of H_2_O_2_ was added, FeO_x_/TiO_2_ presented an outstanding performance as a photocatalyst for the methane oxidation to methanol at room temperature. FeOx/TiO_2_ catalyst showed a high methanol yield of 1056 mmol g^−1^ after 3 h of 300 W Xe lamp irradiation in a batch reactor purged with 70 mmol methane, with roughly 90% selectivity for methanol and 15% methane conversion. Yet, the challenge regarding the low selectivity of oxygenated products in photocatalytic partial oxidation of methane needs to be addressed. This low selectivity is largely due to the fact that the C-H dissociation energy of the oxygenates is lower than that of methane, inevitably resulting in the overoxidation of oxygenates such as methanol to CO_2_. In this review, the photocatalysts will not be elaborated on in detail. This study will be focused on developing catalysts that may directly convert methane to methanol under mild conditions. As reported in the literature, various catalysts, including zeolites, MOFs, and graphene, together with nanomaterials immobilized in these supports, are the most commonly used systems to achieve this goal. These catalysts and their working conditions are presented in Table 1 and Table 2. It can be observed that many novel catalysts use relatively low temperatures. However, maintaining high catalytic activity and methanol selectivity under these mild conditions is a field of present research, and new findings are regularly published.

## 3. Traditional Catalysts

**Figure 3:**
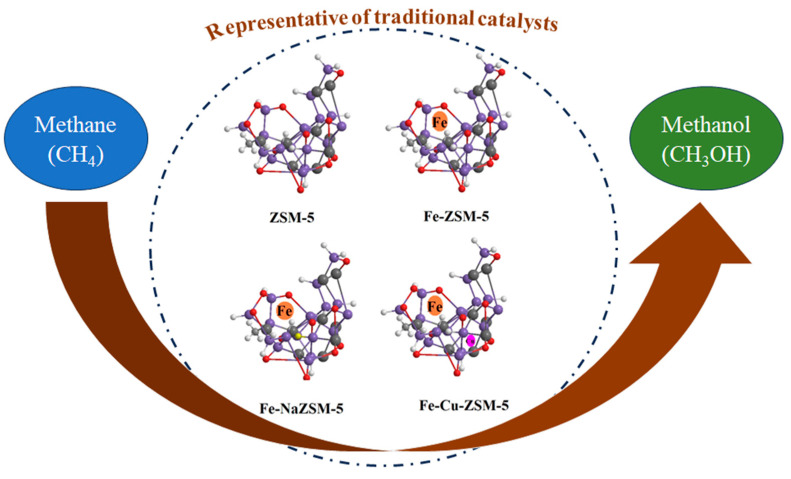
Methane to methanol oxidation over traditional zeolites.

Inspired by the natural methane monooxygenase mechanism in methanotrophic bacteria, zeolites have gained popularity as catalysts for the direct conversion of methane to methanol (Table 1 and Figure 3). In 1997, Kudo et al. investigated the catalytic activity of ZSM-5 as the first zeolites used for the partial oxidation of methane [38]. The maximum selectivity for methanol was not more than 10%, and the major product of the catalysis was carbon dioxide, with a selectivity of more than 80% at 0.01 bar methane partial pressure and 600–700 °C after 1 h. Fe-ZSM-5 is among the pioneer zeolites that have been extensively investigated by researchers during the last two decades for the catalytic conversion of methane to methanol [39,40,41,42,43,44,45,46,47]. Michalkiewicz studied both sodium and hydrogen forms of Fe-ZSM-5 at atmospheric pressure and 350–650 °C using oxygen as the oxidant and achieved 74% selectivity for methanol using Fe-NaZSM-5 [39]. Panov et al. investigated the catalytic activity of FeZSM-5 by increasing the concentration of α-sites at 160 °C and sub-ambient pressure using N_2_O as an oxidant and achieved a methanol yield ranging from 34 to 160 µmol/g_cat_ and a 76 to 95% methanol selectivity [40]. Hammond et al. investigated Fe-containing MIF-type zeolites more deeply and showed that these zeolites can be used for the oxidation of methane at high catalytic rates and high selectivity at mild temperatures in the aqueous phase using hydrogen peroxide as an oxidant [44]. Xu et al. could accomplish iron and copper-modified ZSM-5 catalysts through chemical vapor impregnation, which demonstrated excellent selectivity (92%). In addition, they showed that the catalysts do not deactivate during continuous reactions while maintaining high selectivity [42]. Over the last decade, copper-exchanged zeolites have been the ones that have been more extensively studied [48,49,50,51,52,53,54,55,56,57,58,59]. Lobo et al. investigated the catalytic performance of Cu-SSZ-13 for methanol production using oxygen and nitrous oxide as oxidants at temperatures ranging from 300 to 450 °C and achieved the maximum methanol yield of 13 µmol/g_cat_ at 200 °C when N_2_O was used for peroxidation. They attributed such results to higher concentrations of active species formed by N_2_O at lower temperatures [60]. Tomkins et al. studied the effect of methane activation temperature with oxygen and methane partial pressure on methanol yield in the isothermal cyclic conversion of methane to methanol over Cu-exchanged zeolite at low temperatures [54]. The maximum methanol yield obtained was reported to be more than 100 µmol/g_cat_ at 36 bar and 450 °C. Sushkevich et al. took advantage of water as the oxidant and proved that water molecules played two important roles in the catalytic procedure. Water facilitates the regeneration of active sites and the desorption of methanol while achieving 97% methanol selectivity [25]. Ohyama et al. examined the catalytic performance of several Cu zeolite catalysts using oxygen and water as oxidants at 300 °C for 24 h [53]. Recently, Fang et al. overcame the main obstacle regarding the activation of methane, demonstrating the ability of [Cu_2_(µ-o)]^+2^-ZSM-5 active sites for the activation of methane towards high selectivity to methanol. They investigated the significant role that water plays in enhancing methanol formation as well as the role of chlorine in promoting the production of active sites and facilitating the production of methanol through enhanced desorption [61]. Yu et al. achieved high methanol yields of 431 mol_MeOH_·mol^−1^_Fe_ per hour at low temperatures with 80% methanol selectivity over Cu-Fe(2/0.1)/ZSM-5. They realized that Cu species in these catalysts facilitate the formation of OH radicals, which react rapidly with CH_3_ radicals to form CH_3_OH [48]. In summary, using traditional zeolites, a maximum methanol yield of 5866 µmol/g_cat_ has been obtained with a high (79.7%) methanol selectivity at 50 °C and 30 bar [55].

**Table 1:** Catalytic conditions of methanol yields and selectivity for various traditional zeolites used as catalysts for the conversion of methane to methanol.

Catalyst	Reaction Time (min)	Temp. (°C)	Pressure (bar)	Oxidant	Methanol Yield (µmol/g_cat_)	Selectivity (%)	Side Products	Refs.
ZSM-5	60	600-700	0.01	O_2_	-	10	CH_2_O CO_2_ O_2_	[38]
FeHZSM-5	2.5 s (Contact time)	630	atmosphere	O_2_	-	16.51	CO_2_ HCHO	[39]
FeNaZSM-5	0.5 s (Contact time)	390	atmosphere	O_2_	-	74.37	CO_2_ HCHO	[39]
FeZSM-5	8–165	160	0.1	N_2_O	160 34	76 95	C_2_H_5_OH C_2_H_4_O	[40]
Fe-ZSM-5 (84)	30	50	30.5	H_2_O_2_	74.4	10	HCOOH CH_3_OOH	[44]
ZSM-5 (86)	30	50	30.5	H_2_O_2_	5.55	72	HCOOH CH_3_OOH	[44]
Fe-silicalite-1 (86)	30	50	30.5	H_2_O_2_	65.18	19	HCOOH CH_3_OOH	[44]
Fe-Cu-ZSM-5 (30)	Steady state = 60 min	50	20	H_2_O_2_	81 (µmol g_cat_^−1^ h^−1^)	92.2	CO_2_	[42]
Cu-SSZ-13	60	200	0.3	N_2_O	13.1	24	CO_2_ HCHO	[60]
Cu-MOR	30	200	36	O_2_	56	100	-	[54]
Cu-MOR	30	200	7	H_2_O	0.204 mol/mol_Cu_	97	H_2_O H_2_	[25]
Cu-ZSM-5-Cl	30	50	30	H_2_O_2_ H_2_O	5866	79.93	CH_3_OOH HOCH_2_OOH	[61]
Cu-ZSM-5-N	30	50	30	H_2_O_2_ H_2_O	3216	73.31	CH_3_OOH HOCH_2_OOH	[61]
Cu-ZSM-5-Ac	30	50	30	H_2_O_2_ H_2_O	2851	74.78	CH_3_OOH HOCH_2_OOH	[61]
Cu-Fe(2/0.1)/ZSM-5	30	50	30	H_2_O_2_	431 mol/mol_Fe_	80	HOCH_2_OOH CH_3_OOH CO_2_	[48]

## 4. Nanoparticles-Based Novel Catalysts

**Figure 4:**
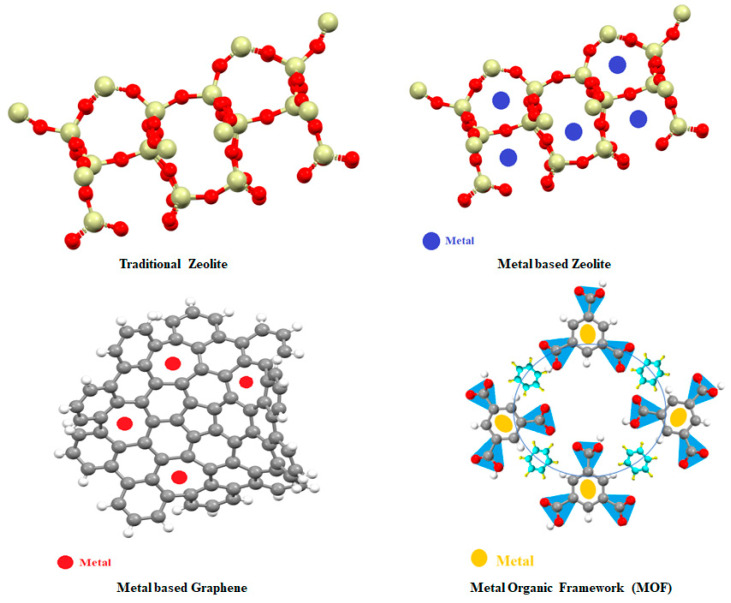
Catalysts based on nanomaterials for methane conversion to methanol.

Metal nanoparticles have gained a strong interest for catalytic purposes during the last few years. However, nanoparticles possess high surface energy, resulting in thermodynamic instability and susceptibility to aggregation during catalytic reactions. To achieve satisfactory performance, critical parameters such as size, shape, and dispersion need to be controlled. In this regard, a variety of surface capping agents such as polyvinylpyrrolidone (PVP), dendrimers, and oleyl amine have been exploited. However, these capping molecules have been shown to attach to metal nanoparticles with very strong interactions that adversely affect the catalytic process. One promising solution to have properly dispersed metal nanoparticles with a clean surface in comparison to traditional zeolite is their incorporation in porous materials such as zeolites, graphene, or MOFs, as shown in Table 2 and Figure 4 [20,21,22].

### 4.1. Nanomaterials Used with Zeolite

Metal nanoparticles have been tried to be loaded on the solid support surface to achieve more efficient heterogeneous catalysts. The solid supports can electronically and geometrically alter the nanoparticles through strong metal-support interactions and provide a high surface area for metal species to disperse [62,63,64]. The majority of the solid supports used so far are Al_2_O_3_, SiO_2_, MgO, ZrO_2_, TiO_2,_ and CeO_2_. However, the supported metal oxide nanoparticles have also demonstrated several negative effects, such as low activity and selectivity. In addition to this, their deactivation can occur due to sintering, leaching, and coke formation under harsh conditions. On the contrary, fixing metallic nanoparticles within zeolite crystals brings the advantage of satisfactory catalytic activity with high selectivity. This happens through several mechanisms. For instance, immobilizing metal nanoparticles within a stable framework such as zeolite would ensure the stability of these metal nanoparticles against sintering and leaching. Additionally, the diffusion of reactant and product can be controlled: the reactant adsorption on the metal nanoparticles can be adjusted, and the reactant and product can be sieved through the pores of the zeolite. When metal nanoparticles are localized in zeolites, their micropores can function as diffusion channels for the reactant and product. This results in shape selectivity [65,66]. So far, very few studies have been carried out regarding nanoparticles supported by zeolite structures for methane-to-methanol conversion. Shan et al. introduced rhodium supported on ZSM-5 zeolite for the oxidation of methane to methanol under mild conditions [67]. In a batch water system with a CH_4_, CO, and O_2_ pressure of 30 bar at 150 °C, this material was tested for catalytic performance evaluation. After an hour, an exceptional methanol yield of 1224 µmol g_cat_^−1^ h^−1^ was obtained. However, the selectivity of methanol was low (8.78%), and the reaction seemed to favor the production of acetic and formic acid. In another similar study, Tang et al. [68] anchored single atoms of rhodium in the micropores of ZSM-5 to convert methane to methanol and acetic acid under low-temperature reaction conditions (150 °C). However, the active sites of the resultant catalyst were more favorable to the production of acetic acid, and therefore the methanol production was very low compared to acetic acid. Lewis et al. supported nanoparticles of gold and palladium on HZSM-5 and used them for the oxidation of methane to methanol under 30 bar methane pressure and at 50 °C of temperature in an aqueous system containing H_2_O_2_ for 30 min and achieved a methanol yield of 51.1 µmol g_cat_^−1^ and relatively low methanol selectivity of 33.6% [69]. Recently, Weng et al. [70] demonstrated that gold nanoparticles dispersed on Mordenite zeolite could selectively catalyze the methane-to-methanol reaction. They achieved an excellent methanol yield of 1300 µmol g_cat_^−1^ h^−1^ with 75% selectivity for methanol. They established that the responsible species for the activation of methane in methanol were both hydroxyl radicals and hydroperoxide species. Therefore, metal nanoparticles loaded on the surface of zeolite proved several advantages, such as improvement of metal sinter resistance and enhancement of selectivity. In addition, the catalysis of metal nanoparticles incorporated into zeolites has the capability of regeneration [71].

### 4.2. Graphene-Based Catalysts

Graphene, a single or a few layers of two-dimensional (2D) *sp*^2^ bonded carbon sheets, possesses a unique structure and extraordinary properties such as high electrical and thermal conductivity, mechanical flexibility, charge-transport mobility, an extremely high surface area, excellent chemical stability, and optical transparency [72,73].

Over the last two decades, graphene has been exploited by scientists for various purposes [74,75,76,77]. Among them, a single individual atom anchored on graphene-based materials has been tested as a novel catalyst since it fulfills the expectations regarding cost-effective catalysis and high surface activity while reducing the use of noble metals. Recently, single metal atoms doped on monolayer graphene surfaces have been used in catalytic reactions for different purposes because of their well-defined site, unsaturated coordination environment, and high atom efficiency [78,79]. Traditionally, supporting noble metal atoms such as Pt and Pd on metal oxides or metal surfaces has been the focus of researchers’ investigation [80,81]. In the case of graphene, Fe, Pd, Pt, Ni, P, and Si are typically dopants that can substitute carbon atoms in graphene sheets to boost their properties [82,83,84,85,86,87,88,89]. In addition, graphene sheets can be tailored by introducing defects in the form of heteroatoms (e.g., N, B, or P) in their structure to accelerate the catalytic reactions occurring on the surface and adjust the electronic properties of the catalysts [82,90,91,92,93,94].

Regarding the conversion of methane to methanol, many materials, such as metal nanoparticles, have been immobilized in different forms of graphene. Despite these advances, the activity and productivity of the methane to methanol seemed to be still dissatisfactory, considering their unique properties, as graphene should be an ideal support. This is what is reported in other applications. Although graphene has been utilized for a great variety of applications, very few studies have been carried out using graphene-based catalysts for methane-to-methanol oxidation. Wang et al. embedded several metal atoms of Co, Mn, Ni, W, and V in graphene based on density functional theory (DFT) calculations and showed that Co atoms enhanced the catalytic performance in comparison to other metals [95]. Impeng et al. [96,97] investigated theoretically the direct oxidation of methane to methanol on Fe-O-modified graphene using N_2_O as an oxidant, with results that were comparable to those of the other previous catalysts. Sanjubala et al. studied the usage of free and graphene-supported single transition metal Cr, Mn, Fe, Co, and Cu atoms for the activation of methane and discovered that Co atoms supported in graphene could be highly effective in the activation of methane [98]. Yuan et al. presented a two-step reaction mechanism for the direct oxidation of methane to methanol on a single atom C-embedded in graphene using N_2_O as an oxidant, and they could conclude that the catalysts would be highly active and would possess good selectivity under mild conditions [99]. Chang et al. exploited DFT to study the catalytic reaction mechanism of methane oxidation to methanol on Bi-functional graphene-oxide-supported platinum nanoclusters. They concluded that this catalyst would have a good performance for the methane-to-methanol reaction and showed that graphene oxide plays an improving role in the catalysis reaction by tuning the interactions between the surface and the adsorbed species [100]. Cui et al. discovered that on the O-FeN_4_-O active sites of graphene-confined single iron atoms, methane can be converted to methanol at room temperature. They showed that the O-FeN_4_-O can activate the C-H bond of methane to form methyl radicals with a very low reaction energy barrier that can be further converted to CH_3_OH and CH_3_OOH [101]. Recently, He et al. studied the direct conversion of methane to methanol on Pd-Au nanoparticles supported on carbon materials such as carbon nanotubes (CNTs), activated carbon (AC), and reduced graphene oxide (rGO) using a gas mixture of oxygen and hydrogen as an oxidant under moderate water aqueous conditions and achieved a methanol productivity of 139 µmol g_cat_^−1^ and a methanol selectivity of 73.2% [102]. Since few studies in this regard have been conducted for methane to methanol oxidation, and some of them are exclusively theoretical, more investigation and experimental studies on graphene utilization as a support for various nano-catalysts to improve the catalytic activity are necessary. Up to date, the best yield obtained using graphene and nanoparticles is 139 µmol/g_cat_ at 50 °C and 33 bar [92].

### 4.3. Nanomaterials Used with MOFs

#### 4.3.1. General Characteristics

MOFs offer considerable opportunities for the incorporation of active sites for catalysis that mimic methane monooxygenases, with high tailorability of the pore structures and environmental conditions in the proximity of the active sites. In this regard, there is already a considerable amount of literature in which various aspects of MOF are well reviewed: synthesis and post-synthetic modifications [103,104,105], active sites and their characterization [106], structure [107,108,109,110], the inclusion of defects [111,112,113,114], water stability [115,116], scale-up of synthesis [117,118,119], multiple functionalities [120,121], application for CO_2_ and biomass conversion [122,123,124,125]. In this review, a deep comparison of the MOF-based catalysts for the conversion of methane to methanol is performed in terms of methane conversion, methanol selectivity, and space-time yield (STY) (Table 2). Most of the published works on MOFs are based on their porous crystalline structure, which can be manipulated in terms of size, geometry, and functionality. The structure of MOFs has been reported to have a high porosity of more than half of the MOF volume. These advantages, together with their high surface area ranging from 1000 to 10,000 m^2^/g, which exceeds that of traditional porous materials such as zeolite and carbon-based materials, make them an excellent candidate for various purposes, especially in catalysis applications [126].

#### 4.3.2. Potentials and Limitations

In general, MOFs offer benefits when used for catalysis. Catalysis by manifold functional groups and also bifunctional or simultaneous catalysis owing to MOFs potential for synthesis and post-synthesis modifications, high catalytic reaction rates per unit volume due to their high internal surface area and active site density, their potential for shape-selective catalysis and having large pores to allow fast transport of product molecules and large reactants due to their pore structure tailorability, and also their Potential for large-scale catalytic applications are among the most significant ones [127,128,129]. Despite these significant advantages, the types of active sites in the structure of MOF are limited, which leads to limited catalytic activity [130]. However, in addition to their inherent active sites, the porous structure of MOFs can be a host for the incorporation of catalytically active sites.

Metal nanoparticles have become more and more interesting for catalytic purposes over the last few years. However, as commented before, nanoparticles have high surface energy, resulting in their thermodynamic instability and susceptibility to aggregation during catalytic reactions. One promising solution to achieve properly dispersed nanoparticles with a clean surface is their incorporation into porous materials [20,21,22]. In this case, MOFs have been the best choice for this purpose. Here, we review the studies that use nanoparticles embedded in MOFs as catalysts for the partial oxidation of methane to methanol. Osadchii et al. incorporated isolated Fe units into Al-based MOF, which successfully imitated the catalytic behavior of the soluble methane monooxygenase (sMMO) enzyme for C-H activation of methane [131]. Through two different synthesis routes, they prepared two different MOF catalysts. The catalytic activity of catalysts was tested under mild conditions in an aqueous environment of water using H_2_O_2_ as the oxidant at temperatures lower than 80 °C for 1 h, leading to highly selective methanol and negligible amounts of overoxidized products such as methyl peroxide, formic acid, and carbon dioxide. Ren et al. proposed the in-situ formation of Cu oxide clusters in UiO-bpy channels and achieved methanol space-time yield and selectivity of 24.33 µmol/g_cat_ and 88.1% with the side product of ethanol under ambient pressure at 200 °C after 3 h. This work included three steps, which were the activation of the catalyst by O_2_, followed by the loading of methane, and finally the extraction of methanol with steam [132]. Xia et al. took good advantage of the combination of catalytic activities of platinum and polyoxometalate via their immobilization into UiO-67 and achieved methanol (12.4%), ethanol (71.3%), and acetic acid (15.9%) under conditions of CH_4_ pressure of 50 bar and temperature of 60 °C after 2 h [133]. They reported 3.5% methanol and 74.9% ethanol after 4 h, which indicates that methanol is oxidized over time. In addition, the low methane conversion was reported to be due to methane’s low solubility in an aqueous solution. Yang et al. introduced an extraordinary MOF-derived mixed hybrid oxide, IrO_2_/CuO, which they synthesized using a bottom-up tactic. Firstly, Ir nanoparticles were synthesized, and then a Cu-containing MOF, Cu-BTC, was utilized as a CuO precursor as well as a host for Ir nanoparticles to be encapsulated to achieve Ir@Cu-BTC, which was further calcinated in the air at 500 °C to produce the final catalyst. IrO_2_ is reported to play a methane activation role, being capable of facilitating the C-H bond cleavage. After the catalysis of methane by this catalyst under the conditions of feeding 3 bar CH_4_/1 bar air at 150 °C after 3 h, they achieved 872 µmol/g_cat_ of methanol. Moreover, they reported a methanol yield of 1937 µmol/g_cat_ when increasing the CH_4_ pressure to 20 bars [134]. Xu et al. loaded AuPd nanoparticles into ZIF-8 and Zn(2-methylimidazole)_2_ to achieve AuPd@ZIF-8 catalyst, and the methanol yield and selectivity were reported at 21.7 µmol g_cat_^−1^ per hour and 21.9% under CH_4_/Ar pressure of 30 bar and an average temperature of 50 °C after 30 min [135]. In addition, the catalytic activity of AuPd@ZIF-8 was compared to the nanoparticles of Au, Pd, and AuPd, as well as Au@ZIF and Pd@ZIF. The earlier comparison well proved the effective role MOFs play in the catalytic performance of the catalyst. Baek et al. synthesized three different MOF catalysts by incorporating three different metal binding ligands into MOF-808 and obtained methanol productivities of 31.7, 61.8, and 71.8 µmol g_cat_^−1^ per hour after methane oxidation at 150 °C for 1 h. The catalysts were reported to have been pretreated with 3% N_2_O/He for 2 h at 150 °C [10]. As reported, at temperatures below 150 °C, methanol was the only product of the methane oxidation, while increasing the temperature seemed to have pushed the methanol to be overoxidized into CO_2_. Moreover, the catalysts appeared to fail in their recyclability, which is attributed to the strong bond that water molecules form with the active sites, which leads to the catalyst’s deactivation. Zheng et al. stabilized Cu-Oxo dimers into NU-1000 MOF for methane oxidation. The catalytic tests for methane to methanol oxidation by this catalyst were carried out at 150–200 °C under pressure varying from 1 to 40 bar and a reaction time range of 30–180 min to observe the effect of contact time, temperature, and pressure on the catalytic activity of the catalyst. As a result, methanol yield and selectivity varied from 1.5 µmol g_cat_^−1^ and 70% (150 °C, 1 bar, 30 min) to 15.81 µmol g_cat_^−1^ and 90% (200 °C, 40 bar, 180 min) [136]. Zheng et al. also used NU-1000 MOF to stabilize Cu-Oxo clusters and used it as a catalyst for methane oxidation. The conditions of the catalytic test were approximately the same, and the results were 17.7 µmol g_cat_^−1^ methanol and 46% selectivity for methanol and dimethyl ether altogether [137]. Hall et al. presented for the first time the roughly exclusive formation of methanol on the Fe^2+^ active sites of MIL-100 (Fe) as a heterogeneous catalyst at mild temperature and sub-ambient pressure with only a trace amount of carbon dioxide produced [138]. In this study, the catalyst was pretreated for 12 h with N_2_O at 250 °C, and then methane and N_2_O were introduced (0.015 bar methane/0.016 bar N_2_O) at 200 °C. Almost every Fe^2+^ site was reported to contribute to the catalytic conversion of methane to achieve a methanol yield of 0.2 µmol g_cat_^−1^. Imyen et al., interestingly, proposed a catalyst by simultaneous exploitation of MOF and zeolites (Fe-ZSM-5@ZIF-8), in which the zeolite is responsible for the methane catalysis while the MOF adsorbs the methane [139]. Primarily, the catalyst was heated at 100 °C to eliminate the surface moisture, and then methane gas (3% CH_4_/He) at 1 bar was fed at 4 mL/min at 50 °C for 2 h to adsorb the methane. Then, the methane feeding was stopped, and the reaction was allowed to continue at 150 °C for the conversion of methane to methanol on the catalyst’s surface for 0.5 h. To collect the produced methanol, the catalyst is said to be flushed with N_2_ (10 mL/min) for 2 h. The methanol was also gathered through steaming, with 40 mL/min of N_2_ bubbling into deionized water at 50 °C. The maximum methanol yield was reported to be 0.12 µmol g_cat_^−1^ when steaming was used for the methanol collection. In summary, using nanoparticles embedded in MOFs, the best yield is 71.8 µmol/g_cat_ at 150 °C. Although metal nanoparticles such as copper and iron-based zeolite can oxidize methane at temperatures ranging from 200 to 600 °C, the product is complex from a gas-phase reaction. Even though the use of platinum-based complexes can oxidize methane in milder conditions, the disadvantages of this type of catalyst are its sensitivity to water and the difficulty of methanol extraction from aqueous solutions. Hence, metal-organic frameworks overcome these problems due to their large surface area, tolerability, porous structures, excellent stewardship as catalysts, and the conversion of methane to methanol at low pressure and temperatures [135].

### 4.4. Other Nanocatalysts

In this section, recent studies supporting nanomaterials, which are not in the last category, are discussed. As mentioned before, supports have been utilized for the single-atom nanocatalysts to enhance the dispersion of the active sites as well as modify the electronic configuration of the nanoparticles, therefore preventing the agglomeration and sintering of the nanoparticles. Additionally, the interface between the metal and the supports has been shown to act as an active site as well. Such interfaces are typically generated as a result of the metal-support interaction. The synergistic function of the different elements present in the composite catalyst is also the underlying reason for the higher catalytic activity of the catalysts. Chen et al. [140] exploited the core-shell nanoparticles of Pd/Pt for the selective oxidation of methane to methanol. The donation of electrons from Pd in the core to Pt in the shell was demonstrated to be the responsible positive parameter for the high rate of methane activation. They obtained a methanol yield of 89.3 mol kg_catalyst_^−1^ h^−1^ with a selectivity of 92.4% at near-ambient temperature values. Gu et al. [141] used atomically dispersed Rhodium (Rh/TiO_2_) for methane oxidation to methanol. In addition, they discovered that using Cu cations as co-catalysts in the solution, higher methanol yields at the millimole level and a selectivity of 99% were obtained. Copper was reported to have played two important roles. The first was that the copper cations maintained the low-valence state of Rhodium and the second was that copper prevented the methanol from overoxidation, leading to high methanol selectivity. It was discussed in the previous sections that overoxidation of methanol has been the main reason for the low values of selectivity as far as methane oxidation is concerned.

**Table 2:** Catalytic conditions and methanol yields and selectivity for metal organic frameworks (MOF) and zeolite used as supports and nanomaterials as active catalysts in the conversion of methane to methanol.

Catalyst	Reaction Time (min)	Temp. (°C)	Pressure (bar)	Oxidant	Methanol Yield (µmol/g_cat_)	Methanol Selectivity (%)	Side Products	Refs.
Rh-ZSM-5	60	150	30	O_2_	1224	8.78	CH_3_COOH HCOOH	[67]
1%Pd/HZS-5 (30)	30	50	30.5	H_2_O_2_	51.1	33.6	CH_3_OOH HCOOH CO_2_	[69]
Au/H-MOR	60	150	30	O_2_	1300	75	CH_3_OOH HCOOH CO_2_	[70]
MIL-53 (Fe, Al)	60	≤60	30.5	H_2_O_2_	-	-	CH_3_OOH CH_2_O_2_ CO_2_	[120]
Cu_x_O_y_@UiO-bpy	180	200	1	O_2_	24	88.1	C_2_H_5_OH	[121]
Uio-67-Pt-Z	120	60	50	H_2_O_2_	-	12.4	C_2_H_5_OH CH_3_COOH	[122]
MOF derived IrO_2_/CuO	180	150	3	H_2_O	872	95	C_2_H_5_OH CH_3_COOH	[123]
AuPd@ZIF-8	30	90	15	H_2_O_2_/O_2_	10.85	21.9	CH_3_OOH HCOOH	[124]
Au@ZIF-8	30	90	15	H_2_O_2_/O_2_	0.7	-	CH_3_OOH HCOOH	[124]
Pd@ZIF-8	30	90	15	H_2_O_2_/O_2_	1.2	-	CH_3_OOH HCOOH	[124]
MOF-808-His-Cu	60	150	-	N_2_O	31.7	100	-	[9]
MOF-808-Iza-Cu	60	150	-	N_2_O	61.8	100	-	[9]
MOF-808-Bzz-Cu	60	150	-	N_2_O	71.8	100	-	[9]
CU-NU-1000	30-180	150-200	1-40	O_2_	1.5–15.81	70–90	C_2_H_5_OH CO_2_	[125]
CU-NU-1000	180	200	1	O_2_	17.7	≤46	C_2_H_5_OH CO_2_	[126]
MIL-100(Fe)	120	200	0.015	N_2_O	0.2	≥98	CO_2_	[127]
Fe-ZSM-5@ZIF-8	300	150	1	-	0.12	-	-	[128]
Pd/Pt core-shell	30	50	30	H_2_O_2_	83 mmol g_cat_^−1^ h^−1^	92.4	CH_3_OOH HCOOH HOCH_2_OOH	[140]
Rh/TiO_2_	60	150	31	H_2_O_2_	-	92		[141]

## 5. Stability and Reusability of Catalysts

An undoubtedly significant issue is the question of the stability and reusability of the catalyst. As observed, in most of the studies in this review, the stability and reusability of the catalyst have not been investigated except for a few works [120,121,123,124,125,127]. Generally, these studies showed good results for long operation times in the range of a few hours. Although this operation time may seem low, it is equivalent to thousands of residence times. However, in industrial applications, the catalyst needs to be stable under the catalytic procedure circumstances for more than one cycle of catalytic reaction in batch mode and longer times in continuous systems while maintaining a good product yield and selectivity. Hence, it is a matter to study in further research, and there is a clear lack of interest in this topic.

## 6. Reactors Used for Methane to Methanol Catalysis

**Figure 5:**
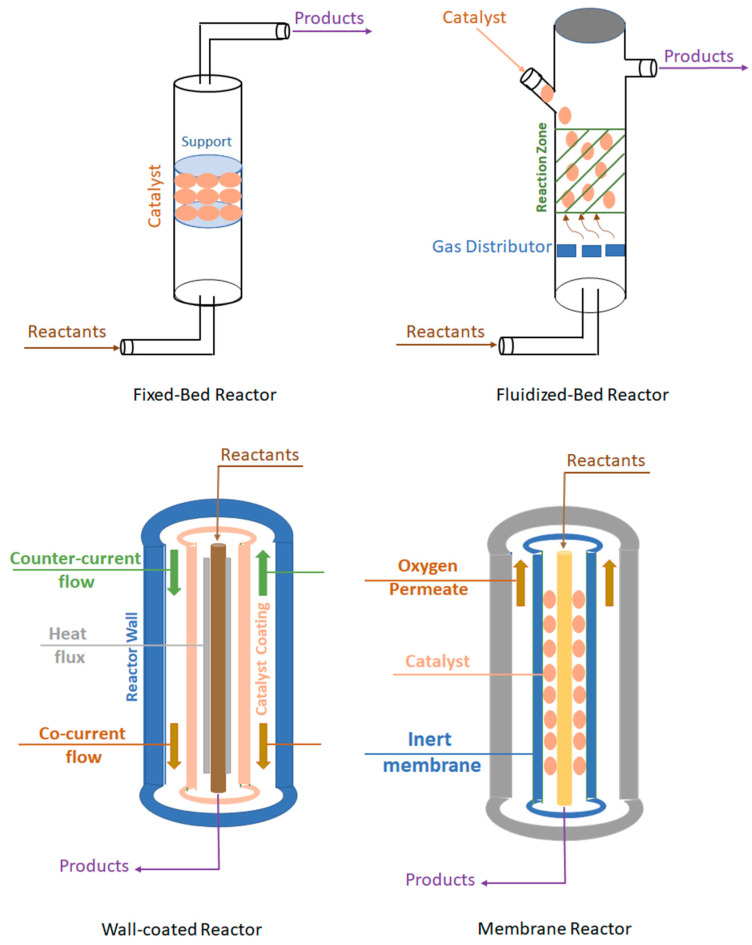
Reactors are used for the catalytic conversion of methane to methanol.

Methane conversion to methanol and valuable products is normally carried out using different types of reactors. The most widely used are fixed-bed, fluidized-bed, well-coated, and membrane reactors, as illustrated in Figure 5.

### 6.1. Fixed-Bed Reactor

A fixed-bed reactor is the most commonly used reactor where a certain amount of the catalyst is fixed in a defined location inside the cylindrical tube of the reactor [142]. This type of reactor can be used in industrial processes as well as for kinetic and catalyst activity studies [143]. Based on one or more catalyst applications in the reactor, this type can be used as a single-stage or multi-stage reactor. In addition, spherical, cylindrical, powdered, or randomly shaped catalysts can be used in this reactor. So, the fixed-bed reactor has benefits such as low cost, high catalyst spatial density, and ease of operation [144]. However, the drawbacks of this type are the drop in high pressure, the low surface area, and the poor distribution of the temperature. Therefore, further studies have been performed in recent decades using the reversal flow mode to improve the capability of heat transfer while maintaining catalyst activity without overheating the catalyst. Recovering the heat from the reversal flow reactor was found to be the most efficient way for methane conversion by optimizing the catalyst bed position, the flow, and the heat exchanger [145].

### 6.2. Fluidized-Bed Reactor

This is also a very common type of catalytic reactor where the catalysts are fluidized during the reaction. The materials inside the reactor are supported by the porous plate; therefore, efficient contact between the catalyst and the reactants is achieved due to the high gas flow. The main benefits of this type of reactor in comparison to the fixed-bed reactor are the uniform temperature distribution and high methane conversion with the increase in temperature [146]. However, the methane conversion seems to decrease by increasing the initial concentration of methane, and an increase in the gas velocity causes the weight loss of the catalyst to increase after the long-term operation [147].

### 6.3. Wall-Coated Reactors

The enhanced mass/heat transfer, lower pressure drops, and increased catalyst contact surface area achieved by depositing a catalyst layer on the reactor wall surface are the main benefits of the so-called wall-coated reactor [144]. Four sub-types of wall-coated reactors are studied in the literature: tubular, monolithic, plate-type, and micro/mini channel plate-type reactors.

#### 6.3.1. Tubular Reactor Type

The performance of this reactor is based on the heat transfer flux, which is normally cold air to remove the release of reaction heat, and the fins are coated with the catalyst that is located at the end of the tube reactor [148]. This design could reach up to 100% conversion of methane when either a 16-finned-tube reactor with high gas velocity or a 10-finned-tube reactor with lower velocity is used. Moreover, the catalytic efficiency of the reactor and the improvement of the diffusion rate of the reactants can be controlled using thinner catalysts and a suitable surface area [149].

#### 6.3.2. Monolithic Reactor Type

The monolithic reactor is suitable for power generation in gas turbines and purification of the emitted pollutants due to its high thermal stability, high rate of mass/heat transfer, and high surface-to-volume ratio [150]. Various types of substrates, such as metallic fibers or foams, and different shapes of the interconnected channels, such as triangles or squares, could be adapted for different applications. For instance, a high specific surface area could be obtained using a monolithic reactor with triangle-interconnected channels [151].

#### 6.3.3. Plate-Type Reactor Type

In this reactor, the co-current and counter-current flow modes occur on the opposite sides of the same plate reactor, combining methane combustion and methane steam reforming reactions. The overlapped temperature zone with the proper co-current mode eliminates hot spots. In addition, the use of folded sheet reactors with rectangular adjacent channels proved the improvement of heat transfer and avoided high heat loss [152].

#### 6.3.4. Microchannel Plate Type Reactor

In the last few years, better catalytic performance has been reported using the microchannel reactor, where the methane conversion takes place on the wash-coated catalyst deposited on the multiple straight channels due to the excellent heat/mass transfer and high surface area of the microchannel [153,154]. Moreover, the reactants can more easily access the inner surface of the microreactor using the porous catalysts that are prepared by the electrodeposition method [155]. The main drawback of this reactor type is the need for extra heat to compensate for the heat loss.

### 6.4. Membrane Reactor

Here, the catalysts are deposited on the surface of the membrane, and this type is one of the most common reactors for methane oxidation due to the efficiency of oxygen permeation, which reacts with methane when it passes through the membrane with air [156]. The efficiency of the reactor depends on the oxygen permeability, flow rate of methane and air, and temperature [157]. Improvement of the methane conversion was conducted using two-pass ion transport, where the oxygen permeation was performed in two stages. In addition, the methane conversion was found to be higher when the configuration of the counter-current flow in this mode of ion transport was used in comparison to the co-current flow configuration [158]. Although high conversion of methane could be achieved using this type of reactor by varying the partial pressure of oxygen permeability, it has limitations in industrial applications due to its high cost.

## 7. Summaries and Perspectives

Methane oxidation to methanol and other value-added chemicals is of high importance in the chemical industry and has gained major interest from researchers. While a great deal of progress has been made in this regard, there are still scientific and technical issues to be addressed. More attempts need to be concentrated to reveal the mechanism of the catalytic reaction and the active sites present in advanced catalysts such as new MOFs and Zeolites-based catalysts. Exploiting the theoretical calculations in conjunction with the development of in situ characterization techniques is highly recommended. In situ characterization studies, such as in situ transmission electron microscopy, can help observe and comprehend the movements and transformations of active sites during the reaction due to harsh reaction conditions. Hence, in the case of confining the metal nanoparticles in porous supports such as MOFs and Zeolites, as we discussed extensively in this review, in situ investigations can reveal the role that such supports play in preventing the active sites from agglomeration and sintering. In addition, mechanistic studies can also reveal the role that the interfaces between the metal and supports play in the activation of methane at lower temperatures. This will provide guidance for the researchers to realize that the incorporation of these supports will lead to better electronic modifications of the metal active sites as well as superior reactive interfacial sites generated, which will serve the methane activation at a lower temperature. On the other hand, different types of MOFs and porous supports need to be investigated to elucidate the potential reactive sites that can be generated as a result of the different metal-support interactions.
